# Simvastatin Combined with Resistance Training Improves Outcomes in Patients with Chronic Heart Failure by Modulating Mitochondrial Membrane Potential and the Janus Kinase/Signal Transducer and Activator of Transcription 3 Signaling Pathways

**DOI:** 10.1155/2022/8430733

**Published:** 2022-03-12

**Authors:** Xiaowen Wang, Kaiyun Yan, Cuifeng Wen, Jiaqi Wang

**Affiliations:** ^1^Department of Neurorehabilitation, Shanghai Third Rehabilitation Hospital, Shanghai 200436, China; ^2^Department of Cardiovascular Medicine, Shanghai Third Rehabilitation Hospital, Shanghai 200436, China; ^3^Tongji University School of Medicine, Shanghai 200092, China

## Abstract

**Background:**

Chronic heart failure (CHF) is the end stage of cardiac disease with a 5-year mortality rate reaching 50%. Simvastatin is an antioxidant with lipid-lowering effects, which is commonly used to treat CHF. Resistance training is a nondrug treatment for CHF and exerts a positive effect on both the myocardial structure and function.

**Objective:**

This study is aimed at exploring the effects and outcomes of simvastatin combined with resistance training on the mitochondrial membrane potential (MMP) of peripheral blood lymphocytes and the Janus kinase/signal transducer and activator of the transcription 3 (JAK/STAT3) signaling pathway in patients with CHF.

**Methods:**

One hundred and eleven patients with CHF were allocated to the control group (CNG) (*n* = 55) and intervention group (IG) (*n* = 56) using the random number table method. The CNG received simvastatin treatment only, whereas the IG received simvastatin treatment plus resistance training. Treatment efficacy, diastolic interventricular septal thickness (IVST), left ventricular ejection fraction (LVEF), left ventricular end-diastolic diameter (LVDD), MMP fluorescence intensity, JAK mRNA and STAT3 mRNA relative expression levels, serum C-reactive protein (CRP), galectin-3, interleukin-6 (IL-6), N-terminal–probrain natriuretic peptide (NT-proBNP), high-sensitivity cardiac troponin T (hs-cTnT), and heart-type fatty acid-binding protein (H-FABP) levels were compared in both groups.

**Results:**

After 6 months of treatment, diastolic IVST, LVDD, and serum levels of CRP, galectin-3, IL-6, NT-proBNP, hs-cTnT, and H-FABP decreased in both groups and were lower in the IG than in the CNG (*P* < 0.05), whereas LVEF, JAK and STAT3 mRNA relative expression levels, and MMP fluorescence intensity of peripheral blood lymphocytes were higher in the IG than in the CNG (*P* < 0.05).

**Conclusion:**

Simvastatin combined with resistance training improves heart function and reduces myocardial damage as well as the occurrence of adverse cardiac events compared with simvastatin alone. The mechanism may be related to the increase of expression of MMP, JAK, and STAT3, the regulation of MMP and JAK/STAT3 signaling pathways in peripheral lymphocytes, the alleviation of mitochondrial damage, and the inhibition of inflammatory response.

## 1. Introduction

Chronic heart failure (CHF) is the end stage of cardiac disease with a 5-year mortality rate reaching 50% [1]. Mitochondria are an important source of energy for the diastolic and contractile functions of the myocardium, and mitochondrial damage is involved in the development of CHF [2]. Mitochondrial membrane potential (MMP) is one indicator that reflects mitochondrial injury in patients with CHF [3]. The activation of the Janus kinase/signal transducer and activator of the transcription 3 (JAK/STAT3) signaling pathway maintains mitochondrial function and plays an important role in various pathological changes, including cardiac hypertrophy, myocardial injury, and ventricular remodeling [4, 5]. Therefore, the detection of MMP fluorescence intensity and JAK/STAT3 expression levels in patients with CHF can help understand disease outcomes and prognosis. Studies have found that MMP and JAK/STAT3 expression levels are noticeably decreased in patients with CHF in contrast to those in healthy controls [6]. MMP and JAK/STAT3 expression levels may serve as serum markers for assessing the recovery status from mitochondrial injury in patients with CHF. Simvastatin is an antioxidant with lipid-lowering effects; it improves left ventricular ejection capacity and protects the myocardium. It is commonly used in the treatment of CHF. Resistance training is one of the nondrug treatments for CHF and exerts a positive effect on both myocardial structure and function. This study explored the effects of simvastatin combined with resistance training on MMP and the JAK/STAT3 signaling pathway in patients with CHF.

## 2. Material and Methods

### 2.1. General Information

A total of 111 patients with CHF treated at our hospital from January 2019 to January 2020 were enrolled. Inclusion criteria were patients who (1) met the diagnostic criteria for CHF in the Chinese Guidelines for the Diagnosis and Treatment of Heart Failure 2018 [7], (2) experienced no episode of acute heart failure in the last month, (3) had no acute cerebrovascular event within the last month, (4) had a history of coronary heart disease, hypertensive heart disease, dilated cardiomyopathy, or pulmonary heart disease, (5) scored II-III on the NYHA Functional Classification, (6) had no communication disorders and had good treatment compliance, (7) had reduced left ventricular ejection fraction (LVEF) or preserved ejection fraction (EF), and (8) signed the written consent form. Exclusion criteria were patients with (1) malignant arrhythmias, aortic stenosis, or atrioventricular block; (2) severe liver, lung, or kidney dysfunction; (3) malignant tumors; (4) psychiatric disorders; (5) acute heart failure; (6) active infections such as hepatitis B, parasitic diseases, and pneumonia; or (7) hematopoietic system diseases and autoimmune diseases. The patients were grouped as control (*n* = 55) and intervention (*n* = 56) groups in accordance with the random number table method. This study was approved by the Ethics Committee of Tongji University School of Medicine.

### 2.2. Methodology

The patients in both the groups received symptomatic treatment with furosemide diuresis and oxygen inhalation. In addition to these, CNG received 20 mg oral simvastatin daily. IG received simvastatin combined with resistance training, with the simvastatin dose being same as that for CNG. Resistance training comprised an exercise performed with a maximum load, i.e., one repetition maximum. The starting load was 50% of the maximum load and increased to 60% in week 9. The resistance training included calf raises; quadricep, tricep, and bicep stretches; shoulder push-ups; abdominal tucks; latissimus dorsi pulldowns; lower back stretches; and chest thrusts. Training forms included weight training, dumbbells, squat raises, and extension springs. The training was supervised and directed by a professional trainer. In the first 4 weeks, 10 repetitions (reps) were performed. At weeks 5–8, 15 reps were performed. During weeks 9–12, only 10 reps each of triceps femoris and biceps brachii stretches, shoulder push-ups, and chest thrusts were performed, followed by 1 min of rest and then 10 reps again. Both the groups were treated for 6 months.

### 2.3. Intervention Indicators

#### 2.3.1. Evaluation of Efficacy

Intervention was considered markedly effective if the NYHA grade improved by more than two grades or returned to normal. Intervention was considered effective if the NYHA grade improved by one grade. Intervention was considered ineffective if the NYHA grade did not improve. The total efficacy was calculated as 100% − ineffective.

#### 2.3.2. Cardiac Function

Interventricular septal thickness (IVST), LVEF, and left ventricular end-diastolic diameter (LVDD) were measured using color Doppler echocardiography before and after treatment.

#### 2.3.3. MMP Fluorescence Intensity

Venous blood (2 mL) was collected before and at 1, 3, and 6 months after treatment. The sample was diluted with 2 mL phosphate-buffered saline (PBS) solution, followed by treatment with 2 mL lymphocyte isolate. Then, diluted anticoagulant solution was added to the sample and centrifuged at 2000 rpm for 20 min. Lymphocytes were aspirated into a centrifuge tube with 2–5 mL of Hank's solution and centrifuged at 1500 rpm for 10 min. PBS precooled in an ice bath was added to resuspend the cells, and a small amount was used for cell counting. The remaining cells were centrifuged at 4°C for 5 min, 1–2.5 mL of mitochondrial isolation reagent was added, and the cells were suspended in an ice bath for 10–15 min. The cells were homogenized into 600 g aliquots and centrifuged at 4°C for 10 min. The cells were then centrifuged in a centrifuge tube at 11,000 rpm at 4°C for 10 min, and mitochondria were obtained via precipitation. Using a fluorescent probe kit, 10 *μ*L of the mitochondrial sample was added and incubated at 37°C for 10 min. The mitochondrial concentration was measured using a luciferase detector with an excitation wavelength of 490 nm and an emission wavelength of 590 nm.

#### 2.3.4. JAK and STAT3 mRNA Relative Expression Levels and RNA Extraction and Purity Assay

Venous blood (2 ml) was drawn and centrifuged at 2000 rpm for 20 min. The serum was decanted and again centrifuged at 2000 rpm for 20 min. The serum was taken and frozen in RNA enzyme tubes. Total RNA was extracted using an miRNA extraction kit and reverse-transcribed to cDNA; glyceraldehyde-3-phosphate dehydrogenase (GAPDH) was used as an internal reference: gapDH (5′-3′)-TTGTGATGGGTGAACCTTCTGAGTGGCAGTGATG, length: 166 bp; JAK (5′-3′)-CCATTTTCTGCACAAACCCACGCCCACAGACAGC, length: 172 bp; STAT3 (5′-3′)-GTAGTGACGGAGAAGCAGTCACAGACTGGTTGTTCC, length: 181 bp. The polymerase chain reaction system comprised of 2 *μ*L 5× buffer, 0.5 *μ*L reverse transcriptase, 0.5 *μ*L oligonucleotides, and 0.5 *μ*L random primers. After adding template RNA, RNase-free water was added to the final volume of 10 10 *μ*g. The reaction conditions were as follows: 37°C for 15 min and 85°C for 5 s. GAPDH was used as the internal reference to calculate the optical density (Δ*Ct*) of JAK and STAT3 miRNAs, and their relative expression levels were calculated using the 2 − Δ*Ct* method.

#### 2.3.5. Inflammatory Markers

Venous blood (3 ml) was collected before and after treatment and was centrifuged in a centrifuge with a radius of rotation of 8 cm at 3000 rpm for 10 min. The supernatant was used to detect CRP levels via the immunoturbidimetric method and galectin-3 and IL-6 levels via an enzyme-linked immunosorbent assay.

#### 2.3.6. Myocardial Injury Markers

Peripheral venous blood (5 mL) was collected before and after treatment, centrifuged at 3000 rpm for 10 min, and the supernatant decanted. Serum N-terminal–probrain natriuretic peptide (NT-proBNP) and high-sensitivity cardiac troponin T (hs-cTnT) levels were detected via chemiluminescent immunoassays, and heart-type fatty acid-binding protein (H-FABP) levels were detected via colloidal gold immunochromatography.

#### 2.3.7. 6 Min Walk Test Distance

A 30 m line was drawn on a flat surface with a mark at every 5 m. The patients were asked to walk 6 m along the line. If symptoms such as fainting, systolic blood pressure > 240 mmHg, diastolic blood pressure > 130 mmHg, blood pressure drop of >20 mmHg, cardiac arrhythmias, and pain appeared, the test was immediately discontinued and the distance was recorded. The test was performed before treatment as well as at 1, 3, and 6 months after treatment.

#### 2.3.8. Adverse Cardiac Events

The patients were observed for the occurrence of any adverse cardiac events during treatment. These events included acute myocardial infarction, persistent myocardial ischemia, cardiogenic shock, and coronary revascularization.

### 2.4. Statistical Analyses

The statistical software SPSS19.0 was applied for data analysis. Measurement data were expressed as $\bar{x}\pm s$ and analyzed using the *t*-test. Count data were expressed as percentages and numbers and analyzed using *χ*^2^ test.

## 3. Results

### 3.1. Comparison of Clinical Data

No significant intergroup differences were observed in age, NYHA classification, proportions of patients with CHF along with diabetes, patients with CHF along with hyperlipidemia, and patients with primary disease (*P* > 0.05; Table 1).

### 3.2. Comparison of Treatment Efficacy

The total efficacy rate was significantly higher in the IG than in the CNG (94.64% vs. 80.00%) (*P* < 0.05), indicating that simvastatin combined with resistance training provides better therapeutic effects than simvastatin alone in patients with CHF (Table 2).

### 3.3. Comparison of Cardiac Function

**Figure 1:**
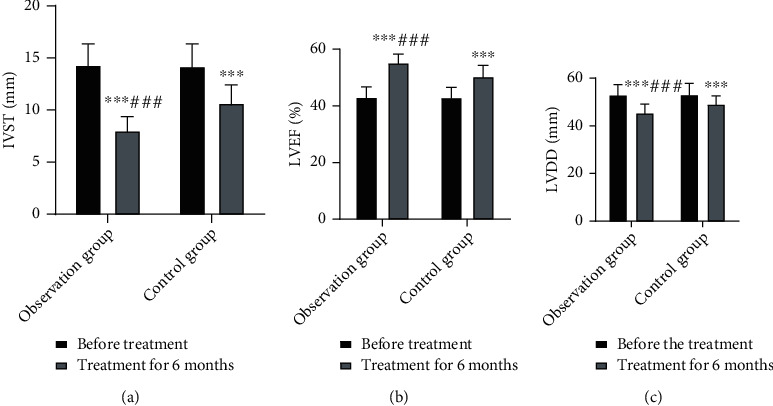
Comparison of cardiac function in both groups. (a) IVST; (b) LVDD; (c) LVEF. Compared with pretreatment, ^∗∗∗^*P* < 0.001; compared with the control group, ^###^*P* < 0.001.

Before treatment, no significant intergroup differences were observed in IVST, LVDD, and LVEF (*P* > 0.05). After 6 months of treatment, IVST and LVDD were noticeably lower, whereas LVEF was remarkably higher than those of pretreatment in both groups (*P* < 0.05). This suggests that simvastatin combined with resistance training significantly improves the cardiac function of patients with CHF (Figure 1).

### 3.4. Comparison of MMP Fluorescence Intensity of Peripheral Blood Lymphocytes

**Figure 2:**
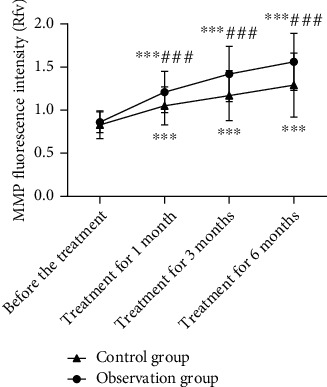
Comparison of mitochondrial membrane potential (MMP) fluorescence intensity of peripheral blood lymphocytes between the two groups (Rfv). Compared with pretreatment, ^∗∗∗^*P* < 0.001; compared with the control group, ^###^*P* < 0.001.

As the treatment continued, MMP fluorescence intensity increased in both groups. It was greater in the IG than in the CNG at 1, 3, and 6 months of treatment (*P* < 0.05; Figure 2).

### 3.5. Comparison of JAK and STAT3 mRNA Relative Expression Levels

**Figure 3:**
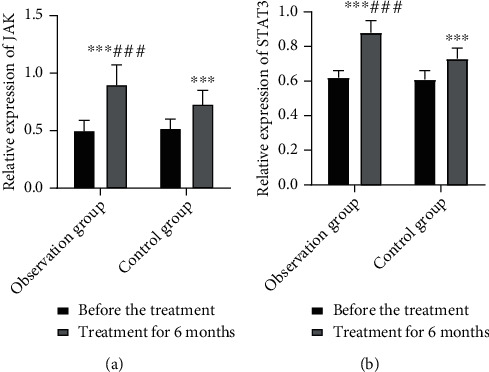
Comparison of JAK/STAT3 relative expression levels in both groups. (a) JAK expression level; (b) STAT3 expression level. Compared with pretreatment, ^∗∗∗^*P* < 0.001; compared with the control group, ^###^*P* < 0.001.

At 6 months after treatment, the relative expression levels in both groups were remarkably higher than those before treatment; furthermore, the IG exhibited remarkably higher relative expression levels than the CNG (*P* < 0.05). This suggests that simvastatin combined with resistance training increases JAK and STAT3 mRNA relative expression levels in patients with CHF (Figure 3).

### 3.6. Comparison of Serum Inflammatory Marker Levels

**Figure 4:**
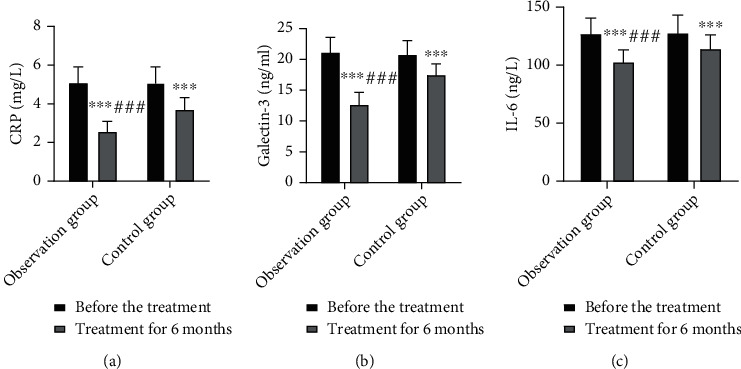
Comparison of serum inflammatory marker levels in both groups. (a) CRP levels; (b) galectin-3 levels; (c) IL-6 levels. Compared with pretreatment, ^∗∗∗^*P* < 0.001; compared with the control group, ^###^*P* < 0.001.

At 6 months after treatment, the levels of the inflammatory markers in both groups were remarkably lower than those before treatment and the IG exhibited significantly lower levels than the CNG (*P* < 0.05). This suggests that simvastatin combined with resistance training significantly reduces serum CRP, galectin-3, and IL-6 levels in patients with CHF (Figure 4).

### 3.7. Comparison of Myocardial Injury Markers

**Figure 5:**
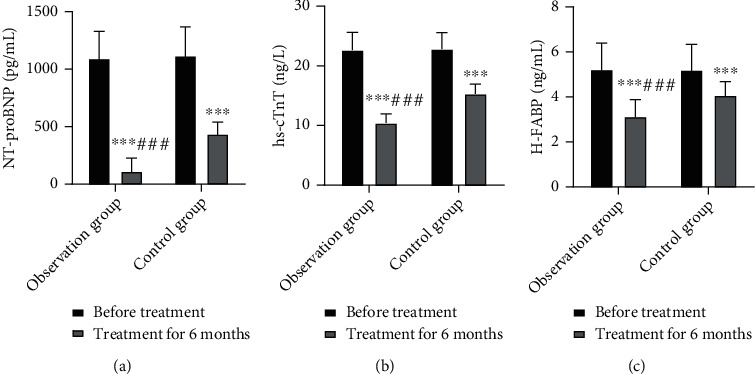
Comparison of myocardial injury markers in both groups. (a) NT-proBNP levels; (b) hs-cTnT levels; (c) H-FABP levels. Compared with pretreatment, ^∗∗∗^*P* < 0.001; compared with the control group, ^###^*P* < 0.001.

At 6 months after treatment, the myocardial injury markers in both groups were significantly lower than before treatment and the IG showed higher levels than the CNG (*P* < 0.05). This finding indicates that simvastatin combined with resistance training significantly reduces serum NT-proBNP, hs-cTnT, and H-FABP levels in patients with CHF (Figure 5).

### 3.8. Comparison of 6-Min Walk Test Distance

As the treatment continued, both groups showed increased trend in the 6-min walk test distance and the distance of the IG was greater than that of the CNG at 1, 3, and 6 months after treatment (*P* < 0.05; Table 3).

### 3.9. Comparison of the Incidence of Adverse Cardiac Events

The incidence of adverse cardiac events was lower in the IG (5.36%) than in the CNG (18.18%) (*P* < 0.05; Table 4).

## 4. Discussion

CHF can lead to alterations in myocardial structure and function, resulting in impaired ventricular function, decreased ventricular ejection capacity, prolonged disease course, and recurrent episodes; it is a leading cause of death in patients with cardiovascular disease [8, 9]. The incidence of CHF in Chinese adults is approximately 0.9%, and it continues to gradually increase [10]. The pathogenesis of CHF is complex, and no treatment is available for this disease. Hence, the primary treatment objective is to improve cardiac function and delay the heart failure process via drug therapy and exercise intervention.

Simvastatin lowers lipid levels and acts on hibernating cardiomyocytes to reverse hibernation, thereby improving left ventricular ejection capacity. It also reduces inflammatory cell chemotaxis and aggregation as well as inhibits matrix metalloproteinase expression by macrophages; thus, it reduces myocardial damage and protects the myocardium. Moreover, it inhibits free radical production and exerts antioxidant effects. Resistance training is one of the intervention therapies for CHF that can increase diastolic, but not systolic, blood pressure, thereby increasing coronary blood flow and improving cardiac function. Resistance training regulates vasoconstrictor system activation, reduces ventricular remodeling, improves myocardial function, slows the heart rate, reduces myocardial perfusion, increases myocardial blood supply, improves patients' exercise endurance, and plays a positive role in improving the long-term prognosis of patients with CHF [11]. The present study results showed that the total efficacy rate of simvastatin combined with resistance training for CHF was 94.64%, which was higher than that of simvastatin treatment alone (80.00%). In addition, IVST and LVDD decreased and LVEF increased with the former, showing superior results over those obtained with simvastatin treatment alone. This suggests that simvastatin combined with resistance training improves the therapeutic effect on patients with CHF as well as improves ventricular remodeling and cardiac function, which is consistent with the findings of an earlier study [12].

Mitochondria maintain the normal activity of cardiomyocytes and provide energy for myocardial activity, and mitochondrial injury is involved in CHF development. The specific mechanism remains unclear, but the current research suggests that mitochondrial injury is one of the pathological mechanisms of the occurrence and development of CHF, and there is an obvious correlation between the degree of mitochondrial injury and the grade of cardiac function [13, 14]. The mitochondrial permeability transition pore is normally closed, but it opens when mitochondrial injury occurs, leading to a decrease in MMP. Therefore, a decrease in MMP indicates mitochondrial injury and is an important marker in detecting mitochondrial damage [15, 16]. The IL-activated JAK/STAT3 signaling pathway is involved in the development of CHF, and the activation of this pathway mitigates oxidative stress injury, inhibits MMP dissipation, and protects mitochondrial function [17]. STAT3 is a key intracellular signal transduction protein and transcription factor, and IL-6 and IL-11 are involved in the transmission of cellular signals to STAT3 by the binding of cell membrane receptors to ligands, causing conformational changes, and forming homodimers or heterodimers [18]. JAK/STAT3 signaling pathway activation inhibits the reduction and dissipation of MMP, reduces mitochondrial damage, and protects the myocardium [19]. Moreover, the pathway modulates electron transport chain enzyme activities and protects mitochondria. Mitochondrial injury in the cardiomyocytes of patients with CHF leads to the accumulation of a large amount of reactive substances, leading to the inhibition of JAK/STAT3 signaling pathway activation [20]. The present study results showed that the MMP fluorescence intensity of peripheral blood lymphocytes and JAK and STAT3 mRNA relative expression levels increased in both groups after treatment; however, the levels were higher in the IG than in the CNG. This finding suggests that simvastatin combined with resistance training increases MMP and JAK/STAT3 expression levels, ameliorates mitochondrial damage, and promotes JAK/STAT3 signaling pathway activation, which may be an important mechanism for improving the condition of patients with CHF.

Multiple inflammatory factors are involved in the pathophysiological process of heart failure, and myocardial injury in heart failure is closely related to inflammation [21, 22]. CRP and IL-6 are common inflammatory mediators that are mainly produced by activated macrophages, whereas galectin-3 is a newly discovered inflammatory marker of heart failure and is produced by macrophages [23]. CHF is a systemic inflammatory disease; individuals with CHF exhibit markedly increased levels of inflammatory cytokines in the blood, which are derived from the myocardium itself as well as circulating platelets, hepatocytes, and leukocytes [24]. NT-proBNP, hs-cTnT, and H-FABP are important markers that indicate cardiomyocyte injury. NT-proBNP is a peptide that is mainly derived from ventricular myocytes [25]. cTnT is a regulatory protein produced by myocardial tissue; it is released during cardiomyocyte necrosis, leading to elevated serum hs-cTnT levels. H-FABP is a newly discovered small-molecule marker that is predominantly found in myocardial tissue; it promotes the release of H-FABP into the blood when myocardial cells are damaged [26]. The present study results showed that after treatment, serum CRP, galectin-3, IL-6, NT-proBNP, hs-cTnT, and H-FABP levels decreased in IG and were lower in this group than in CNG. These findings suggest that simvastatin combined with resistance training reduces inflammatory responses and ameliorate myocardial damage in patients with CHF. Furthermore, the study results showed that the 6 min walk test distance was greater in IG and that fewer adverse cardiac events were observed in this group. This finding suggests that simvastatin combined with resistance training improves patients' mobility and reduces adverse cardiac events.

## 5. Conclusion

In summary, simvastatin combined with resistance training improves the therapeutic effect, improves cardiac function, and reduces myocardial injury as well as adverse cardiac events in patients with CHF. However, this study has some shortcomings, such as a small sample size and a short follow-up period. Therefore, it is necessary to evaluate a larger group of patients to validate the value of simvastatin combined with resistance training in the prognosis of patients with CHF.

## Figures and Tables

**Table 1:** Comparison of baseline data of both groups [$\bar{\text{x}}\pm \text{s}$, n (%)].

Grouping	*n*	Gender		Average age (years)	NYHA classification		Combined with diabetes	Combined with hyperlipidemia	Primary disease			
		Male	Female		II	III			Coronary heart disease	Dilated cardiomyopathy	Hypertensive heart disease	Pulmonary heart disease
Intervention group	56	33 (58.93)	23 (41.07)	62.61 ± 5.11	35 (62.50)	21 (37.50)	15 (26.79)	22 (39.29)	21 (37.50)	13 (23.21)	12 (21.43)	10 (17.86)
Control group	55	30 (54.55)	25 (45.45)	61.94 ± 5.70	37 (67.27)	18 (32.73)	17 (30.71)	20 (36.36)	20 (36.36)	15 (27.27)	10 (18.18)	10 (18.18)
*χ* ^2^/*t*		0.217		0.652	0.277		0.230	0.101	0.340			
*P*		0.641		0.516	0.599		0.632	0.751	0.952			

**Table 2:** Comparison of the efficacy of both groups [%(*n*/*n*)].

Grouping	*n*	Marked efficiency	Effective rate	Ineffective rate	Total effective rate
Intervention group	56	62.50 (35/56)	32.14 (18/56)	5.36 (3/56)	94.64
Control group	55	45.45 (25/55)	34.55 (17/55)	20.00 (11/55)	80.00
*χ* ^2^					5.398
*P*					0.020

**Table 3:** Comparison of 6-minute walking distance of both groups ($\bar{x}\pm s$, m).

Grouping	*n*	Pretreatment	1 month of treatment	3 months of treatment	6 months of treatment
Intervention group	56	185.36 ± 22.17	246.35 ± 20.10^∗∗∗###^	275.02 ± 21.38^∗∗∗###^	292.46 ± 20.35^∗∗∗###^
Control group	55	181.21 ± 23.06	207.67 ± 23.27^∗∗∗^	236.47 ± 22.23^∗∗∗^	255.38 ± 22.62^∗∗∗^
*t*		0.967	9.377	9.313	9.083
*P*		0.336	0.001	0.001	0.001

Compared with pretreatment, ^∗∗∗^*P* < 0.001; compared with the control group, ^###^*P* < 0.001.

**Table 4:** Comparison of adverse cardiac events of both groups [*n* (%)].

Grouping	*n*	Acute myocardial infarction	Intractable myocardial ischemia	Cardiogenic shock	Coronary blood flow reconstruction	Cardiac death	Incidence rate
Intervention group	56	1 (1.78)	1 (1.78)	0 (0.00)	1 (1.78)	0 (0.00)	5.36 (3/56)
Control group	55	4 (7.27)	2 (3.64)	1 (1.82)	2 (3.64)	1 (1.82)	18.18 (10/55)
*χ* ^2^							4.414
*P*							0.036

## Data Availability

This manuscript has no associated data.
